# Design Method of Three-Component Optic Fiber Balance Based on Fabry–Perot Displacement Sensor

**DOI:** 10.3390/s23177492

**Published:** 2023-08-29

**Authors:** Bin Xu, Shien Yu, Jianzhong Zhang

**Affiliations:** 1 Key Laboratory of In-fiber Integratd Optics of Ministry of Education, College of Physics and Optoelectronic Engineering, Harbin Engineering University, Harbin 150000, China; 2 Hypervelocity Aerodynamics Institute, China Aerodynamics Research and Development Center, Mianyang 621000, China

**Keywords:** pulse wind tunnel, aerodynamic measurement, Fabry–Perot displacement sensor, optic fiber balance, finite element simulation, static calibration

## Abstract

This article proposes a new type of three-component optic fiber balance based on Fabry–Perot displacement measurement technology based on the structure of the pulse wind tunnel balance. This paper systematically introduces the force measurement principle and design process of a three-component optic fiber balance and conducts relevant simulation analysis and experimental verification. The simulation results show that the Fabry–Perot sensor can achieve significant sensitivity to cavity length changes, and when used in existing balance structures, sensitivity gains can be achieved by changing the probe height without the need to modify the original structure of the balance. Finally, the feasibility of the design method was verified through calibration experiments: the optic fiber balance has high sensitivity and good linearity compared to simulation sensitivity, the error is less than 6%, and the calibration accuracy of each component is better than 0.13%, which is better than the existing traditional strain balance (0.37%). The pulse wind tunnel force measurement test has a short test time and a large model mass, and the balance needs to have a large stiffness to meet the short-term force measurement requirements. The introduction of more sensitive optic fiber balance force measurement technology is expected to solve the contradiction between the stiffness and sensitivity of force measurement systems.

## 1. Introduction

Ground wind tunnel testing is the most important means for conducting research on the aerodynamic characteristics and various performances of aircraft [1]. Among numerous types of wind tunnel equipment, pulse wind tunnel equipment has the characteristics of a simple system, low construction and operation costs, and convenient maintenance, making it one of the most important pieces of equipment for conducting aircraft testing research [2,3]. In wind tunnel test projects, force testing is one of the most basic test items, mainly measuring the magnitude, position, and direction of the load on the aircraft during the test process. However, due to the short operating time of the pulse wind tunnel (on the order of one hundred milliseconds), in order to obtain more accurate aerodynamic loads and the thrust generated by the engine, the entire force measurement system needs to have a high frequency response and fast response speed [4,5,6,7]. At present, pulse wind tunnel force testing mainly relies on traditional strain balance force measurement technology, which measures the strain of the balance structure when the model is subjected to load using a Wheatstone bridge to obtain the force state of the model. After long-term development and research, as well as experimental results, it has been shown that the strain balance force measurement technology has high measurement accuracy and can better meet the current force measurement needs [8,9,10]. However, with the continuous improvement of experimental requirements, the size of the model will increase, and the weight of the model will also increase. However, due to the limited sensitivity of traditional strain balance, in order to ensure the load resolution ability of the system, there are certain requirements for system stiffness, which will greatly reduce the frequency response of the existing strain balance force measurement system. Therefore, in the face of the force measurement needs of larger-scale models, it is necessary to introduce new force measurement technologies to improve the sensitivity of the force measurement system and solve the contradiction between the sensitivity and stiffness requirements of the existing pulse wind tunnel force measurement system.

In recent years, with the development of optic fiber sensing technology, it has been widely used in aerospace, especially in the field of wind tunnel testing, due to its high sensitivity, resistance to electromagnetic interference, and high temperature resistance [11,12,13,14]. The optic fiber balance force measurement technology based on optic fiber sensing technology has quickly become a research hotspot in the field of wind tunnel force measurement. Through wind tunnel experiments, it has been proven that optic fiber balances have significant advantages over traditional strain balances in harsh environments such as high temperatures and strong electromagnetic radiation [11,15]. Based on this, in order to improve the sensitivity and measurement accuracy of the balance, Pieters et al. [16,17] applied the concept of “Two-groove OFBG” to design a six-component optic fiber balance based on fiber Bragg grating (FBG) and conducted simulation analysis. The simulation results show that the balance using this design concept has advantages such as simple structure, good stiffness, high sensitivity, and small interference from each component. In addition to FBG, Fabry–Perot (F-P) strain gauges have also been applied in optic fiber balances. Qiu et al. [18,19] used micro-opto-electro-mechanical F–P strain sensors and all-fiber F–P strain gauges to produce a three-component wind tunnel balance. The experimental results show that the static performance of optic fiber balances is superior to that of traditional strain balances. However, at present, the application field of optic fiber balance is mainly in conventional continuous wind tunnels. Compared with pulse wind tunnel force testing, the two types of wind tunnels have different operating characteristics. The continuous wind tunnel can operate for hundreds of seconds, and the differences in test models result in different design methods for balance structures. Different wind tunnel force balances need to choose balance structures and appropriate sensing schemes based on the characteristics of wind tunnel operation and force measurement requirements.

This article designs a three-component optic fiber balance based on Fabry–Perot displacement measurement. The first part of the article mainly elaborates on the difficulties faced in the design of pulse wind tunnel balance and the current research status of optic fiber balance. The second part systematically introduces the design method of the Fabry–Perot displacement sensor and the three-component optic fiber balance. The design parameters of the F–P displacement sensor were obtained through simulation analysis, and the measurement of the three-component aerodynamic load and temperature self-compensation were achieved by arranging eight F–P displacement sensors. The third part tested the performance and temperature compensation characteristics of the optic fiber balance, and the experimental results showed that the three-component load outputs of the optic fiber balance were basically consistent with the simulation results and superior to existing strain balances. The optic fiber balance force measurement technology provides a new technical route for improving the quality of pulse wind tunnel test data and expanding test capabilities.

## 2. Three-Component Optic Fiber Balance Based on Fabry–Perot Displacement Measurement Sensor

### 2.1. Structure and Principles of Optic Fiber Balance

**Figure 1:**
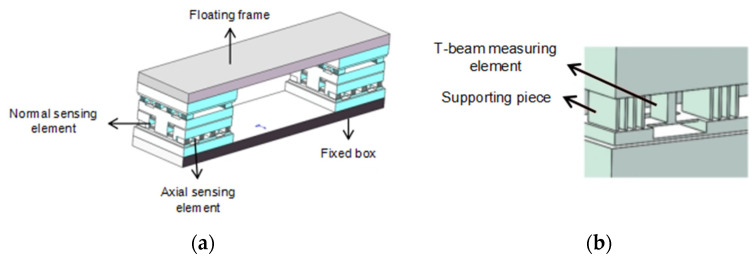
The structure of optic fiber balance. (**a**) The overall structure of the balance. (**b**) Balance sensitive elements.

The structure of the optic fiber balance is shown in Figure 1a, consisting of a balance floating frame, a sensitive element, and a fixed frame. During the force measurement process, the floating frame of the balance is connected to the model, and the fixed frame is fixed to the bottom support platform. When the model is subjected to aerodynamic loads, the sensitive element will deform. By measuring the deformation of the sensitive element, the load on the model can be obtained. The sensitive elements of the balance are shown in Figure 1b, each of which is composed of a T-shaped measuring element and multiple support plates. The T-shaped measuring element is only sensitive to the load perpendicular to the vertical beam, and the support plate is used to improve the overall stiffness of the balance. The balance structure designed in this article is composed of vertically connected sensitive components that are sensitive to the load in three directions, achieving mechanical decomposition of loads in different directions structurally.

### 2.2. Principle of Fabry–Perot Displacement Measurement

**Figure 2:**
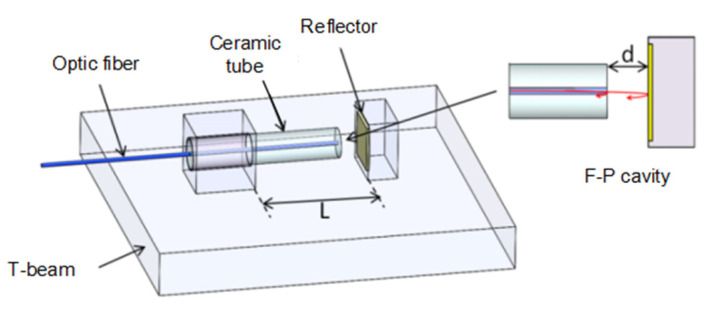
Structural schematic diagram of the F–P displacement sensor.

The schematic diagram of the Fabry–Perot displacement sensor is shown in Figure 2. The Fabry–Perot cavity is composed of a fiber end face and a rear end reflection surface. According to the principle of Fabry–Perot interference, when light is incident into the Fabry–Perot cavity, multiple reflections will occur in the F–P cavity, resulting in a multi-beam interference effect. When the incident light intensity is $I_{i}$, regardless of the transmission loss of light in the cavity, the reflected output light, $I_{r},$ can be expressed as [20]:

(1) $$I_{r}=\frac{4R\sin^{2}(\frac{\varphi }{2})}{{\left(1-R\right)}^{2}+4R\sin^{2}(\frac{\varphi }{2})}I_{i}$$

where $I_{i}$ is the radiation intensity, *R* is the reflectivity of the reflecting surface, and φ is the interference phase, which is calculated by:

(2) $$\varphi =\frac{4\pi nd}{\lambda }+\varphi_{0}$$

where *d* is the length of the F–P cavity, λ is the wavelength of the incident light, *n* is the refractive index of the F–P cavity medium, and $\varphi_{0}$ is the initial phase.

When the reflectivity of the reflector composed of an F–P cavity is low (the reflectivity of the fiber end is about 4%), only two beams of reflected light need to be considered. Therefore, the reflected light intensity $I_{r}$ of Equation (1) can be simplified as:

(3) $$I_{r}=I_{1}+I_{2}+2\sqrt{I_{1}I_{2}}\cos\varphi$$

When the light source is broadband light, the typical spectrum of the interference between two beams of light approximates a cosine function, as shown by Equation (3). The maximum reflected light intensity occurs when φ = 2*mπ* (*m* = 0, 1, 2). Due to the fact that the interference phase of two beams of light depends on the F–P cavity length, the reflection spectrum will drift when the cavity length changes, which can be used for strain and displacement sensing. When the structure undergoes deformation, the relationship between the output value of the F–P sensor and the strain applied can be expressed as:

(4) $$\Delta \lambda =\lambda_{0}\cdot \frac{L\varepsilon }{d}=\lambda_{0}\cdot \frac{\Delta L}{d}$$

where $\lambda_{0}$ and Δ*λ* are the initial peak wavelengths of the F–P sensor and the wavelength drift caused by strain, respectively, and *L* is the sensing length, respectively. In the F–P displacement sensing structure shown in Figure 2, the sensing length *L* is the preset distance between displacement measurement points. In traditional F–P strain gauges, the cavity length is generally the sensing length. In this displacement sensing structure, the sensing length *L* of the sensor can be much larger than the cavity length d; therefore, under the same deformation of the structure, a higher sensitivity output can be achieved.

### 2.3. Simulation Analysis and Sensor Parameter Optimization

The T-beam measuring element used in the structure of the wind tunnel balance is sensitive to loads perpendicular to the beam direction. During the force measurement process of the balance, the vertical beam of the T-shaped element will bend, and its surface will generate axial non-uniform tension and compression. This makes the selection of displacement measurement points directly affect the displacement change value under the same load. At the same time, bending will create an angle between the reflection surfaces, causing a deviation between the measured displacement and the theoretical axial displacement. Even when the angle is large, it will increase the bending loss in the F–P cavity, making it impossible to form effective interference. To solve this problem, the deformation characteristics of the measuring element of the balance under load are analyzed by COMSOL finite element simulation. The balance material is maraging steel F141; the material parameters are shown in Table 1, and the mesh is a tetrahedral mesh that comes with COMSOL finite element analysis software. According to the working principle of T-beam components, fix the C and D ends and apply normal displacement at the A end. The displacement is the measured load divided by the design stiffness of the balance in that direction.

**Figure 3:**
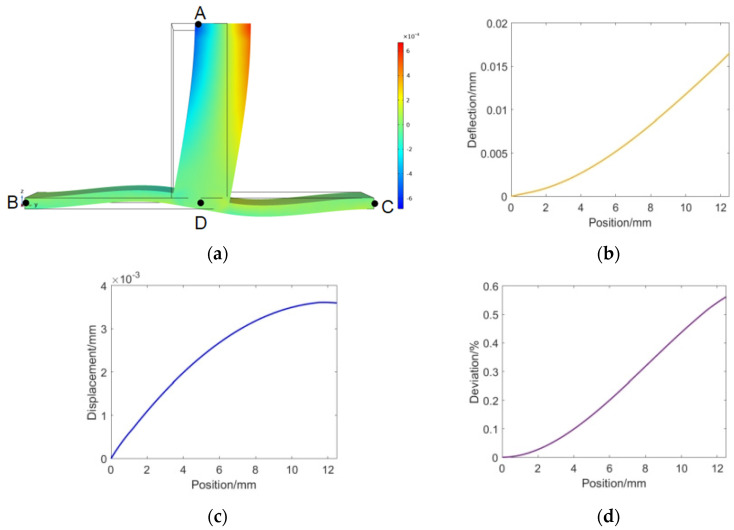
Simulation analysis results of the T−beam. (**a**) Diagram of T−beam deformation. (**b**) Axial deflection distribution of section AB. (**c**) Axial displacement distribution of the AB section. (**d**) Measurement deviation caused by bending of the beam.

The deformation of the T-beam under a sensitive load is shown in Figure 3a. The free end A has horizontal displacement, C and D are fixed ends, and the AB section produces single curvature deformation during stress, which is used for sensor layout (the length of the AB section is 12.5 mm). The axial displacement and deflection distribution of each point on the AB section are shown in Figure 3b,c. From the axial displacement distribution of beam structure, it can be seen that when the initial spacing L of sensor displacement measurement points is larger, the sensor sensitivity is higher, and at the same sensing length, the closer the measuring point is to the free end A, the higher its sensitivity will be. Secondly, due to the variation of the bending degree of the beam structure with the load, the deviation generated during the force measurement process is also nonlinear and difficult to correct. Therefore, the maximum deviation value is required, which means that under full range loading, the deviation value is less than the design requirements of the balance. Figure 3d shows the deviation between the actual cavity length change and the theoretical value when fully loaded. From the simulation results, it can be seen that although the larger the sensing length L, the higher the sensitivity of the cavity length change, it will also increase measurement errors. It is necessary to consider both the sensitivity requirements of the balance and the measurement accuracy requirements.

**Figure 4:**
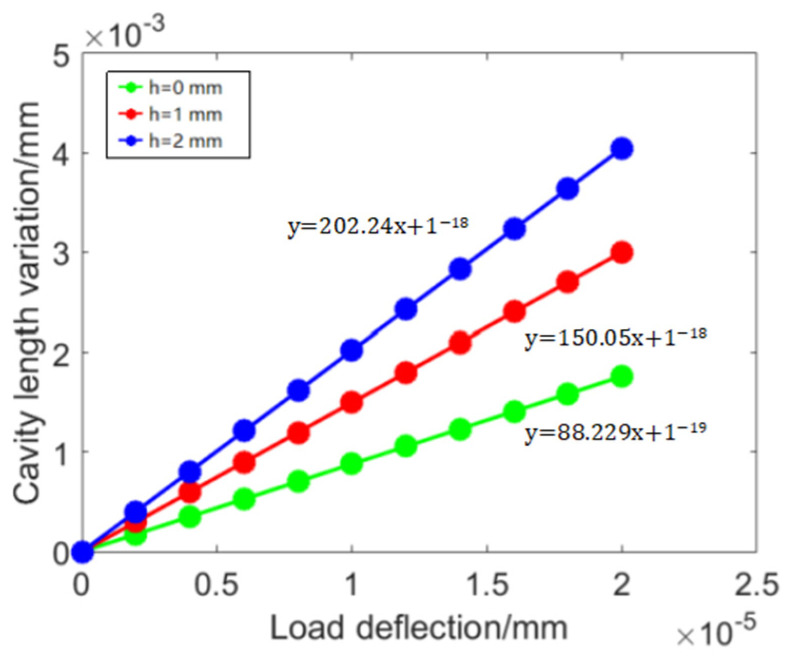
The displacement measured by the sensor at different *h*.

In addition to the sensing length *L*, the simulation results show that the height *h* of the optic fiber is also the main factor affecting the static performance of the sensor. When *L* is 3 mm, a horizontal displacement is applied to point A, and the variation of the axial displacement measured by the sensor with *h* is shown in Figure 4. The higher the height of the optic fiber, the greater the displacement; moreover, changing the height *h* of the probe does not affect the angle between the reflecting surfaces, so it can be achieved within the range of the balance. Increasing the probe height *h* not only improves the sensitivity of the sensor but also does not result in an increase in measurement deviation.

### 2.4. Design of a Three-Component Optic Fiber Balance

**Figure 5:**
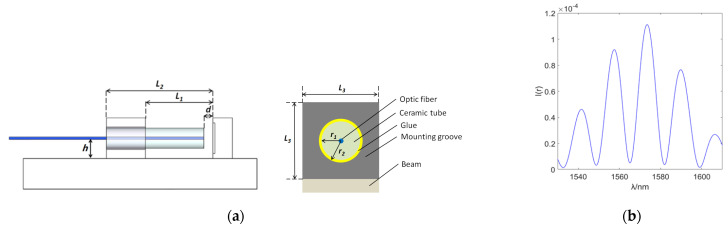
F−P displacement sensor and its output spectrum. (**a**) A 2−D structure diagram of the sensor. (**b**) Sensor reflection spectrum.

The structure of the F-P displacement sensor is shown in Figure 5a. Based on the balance design requirements and simulation analysis results, the final sensor design parameters are shown in Table 2. The manufacturing process is as follows. Firstly, to protect the optical fiber and prevent fiber bending, a ceramic tube is used to encapsulate the single-mode optical fiber. Due to the low coefficient of thermal expansion of optical fiber materials and ceramic materials, the temperature effect of the sensor can be avoided as far as possible. Secondly, the optical fiber installation slot is processed at a position 3 mm from the beam root, the packaged optical fiber is inserted into the installation slot, and the reflector is installed in the vertical plane with the root of the T-beam. The optical fiber installation slot is drilled in advance, and the aperture matches the external diameter of the ceramic packaging tube to ensure the stability of the sensor after installation. After inserting the optical fiber into the installation slot, the F–P cavity length d can be adjusted by moving the fiber end back and forth through a micro-displacement device. During this process, observing the reflection spectrum of the sensor can monitor the changes in cavity length d. After adjusting the cavity length, fix the encapsulated optical fiber with epoxy resin adhesive. The F–P sensor has a simple structure, high sensitivity, and does not require the application of pre-stress during installation, avoiding the impact of pre-stress relaxation on sensor performance. The reflection spectrum of the F–P displacement sensor after production is shown in Figure 5b.

**Figure 6:**
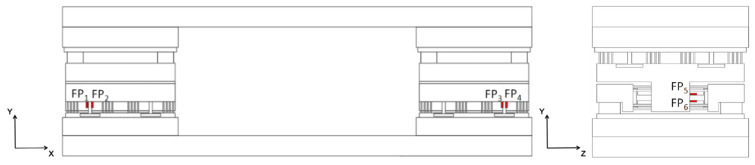

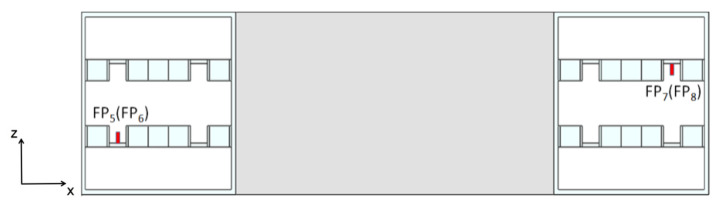
Mounting positions of the eight sensors.

The loads measured by the three-component optic fiber balance are the axial force (F_A_), normal force (F_N_), and pitch moment (M_z_) on the aircraft or spacecraft model. The installation positions of the eight F–P displacement sensors used in the balance are shown in Figure 6. Based on the working principle of the balance and the deformation characteristics of the sensitive components under different loads, the sensors are installed on four T-beam measuring elements, and each sensitive element is symmetrically arranged with F–P displacement sensors; this is mainly for temperature compensation. Since the deformation trend of the upper and lower symmetrical planes of the T-beam structure is opposite under the aerodynamic load (one plane is stretched, the other is compressed), and the positive and negative trends of the output caused by the thermal output of the sensor itself and the thermal expansion of the structure are the same, the temperature self-compensation can be realized by the way of bridge assembly output by arranging the sensor in the symmetrical position of the beam structure. The outputs of two sensors symmetrically arranged in the T-beam measuring element can be represented as:

(5) $$\left\{ \begin{array}{l} \Delta \lambda_{1}=K_{1}(\varepsilon_{F}+\varepsilon_{\Delta Τ})+K_{2}\Delta Τ\\ \Delta \lambda_{2}=K_{1}(-\varepsilon_{F}+\varepsilon_{\Delta Τ})+K_{2}\Delta Τ\\ \Delta \lambda_{F}=\Delta \lambda_{1}-\Delta \lambda_{2} \end{array}\right.$$

where $\varepsilon_{F}$ and $\varepsilon_{\Delta T}$ the strain of the balance structure caused by aerodynamic load and thermal expansion is respectively represented. When the design parameters of the sensors are basically the same, the strain and temperature sensitivity coefficients ($K_{1}$, $K_{2}$) of the two sensors can be considered approximate. When the temperature at each position of the balance structure is the same, the thermal output of the balance can be self-compensated by the difference between the outputs of the two sensors. Finally, the decoupling formula for its three-component load is shown in Table 3.

## 3. Calibration Test of Optic Fiber Balance

### 3.1. Temperature Compensation Test

**Figure 7:**
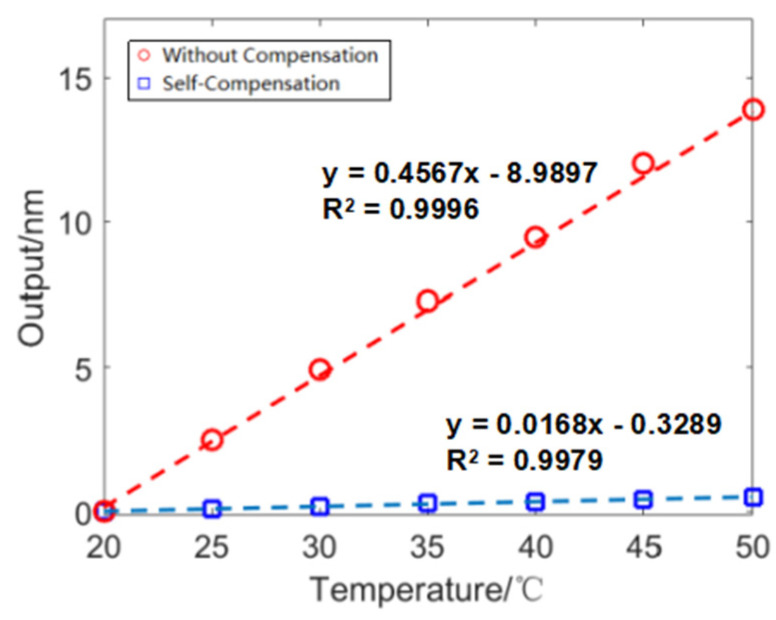
Temperature sensor calibration.

The compensation of the thermal output of wind tunnel balance has always been an important issue in balance force measurement technology. In wind tunnel experiments, the force balance is generally placed inside the model to prevent it from being affected by external heat flux. However, in conventional continuous wind tunnel aerodynamic tests, due to the long test time, the model and support rods are affected by the airflow and transfer heat to the wind tunnel balance, resulting in an increase in the temperature of the balance and sensors, resulting in thermal output. The pulse wind tunnel test time is less than 1 s, and the model size is large. During the wind tunnel test, the temperature transfer effect was relatively small. However, during the long-term calibration process, changes in environmental temperature can also cause thermal strain on the balance and the thermal output of the sensor. To ensure the accuracy of subsequent static calibration tests and evaluate the compensation effect of temperature self-compensation methods, temperature calibration tests were first conducted, and the calibration results are shown in Figure 7. After self-compensation, the temperature sensitivity decreases by about 97.4% compared to a single F–P sensor. When the bridge is output, the temperature sensitivity of each component of the balance is shown in Table 4. The compensation accuracy of the balance’s three-component thermal output is higher than 93.3%. At the same time, according to the balance output formula, the aerodynamic load output is four times the output value of a single sensor, further reducing the proportion of the balance’s thermal output.

### 3.2. Calibration Device and Testing

**Figure 8:**
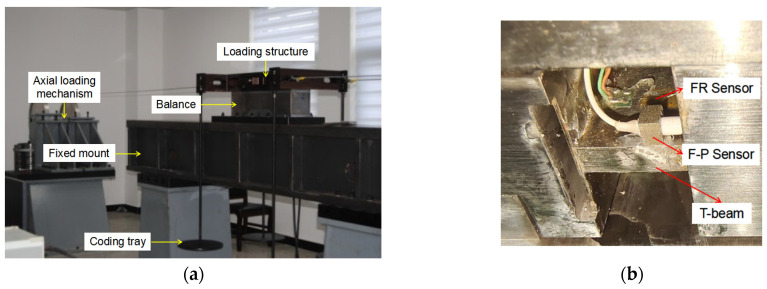
Optic fiber balance calibration test: (**a**) balance calibration device and (**b**) sensor layout.

In order to accurately measure the aerodynamic force during wind tunnel testing, the balance needs to be calibrated to obtain the relationship between load and balance output. The static calibration of the balance is carried out using the calibration device shown in Figure 8a. Firstly, fix the balance on the supporting platform and install a loading structure on the floating frame of the balance. By adding weights, loads from different directions can be transmitted to the balance. The calibration of the optic fiber balance uses unit calibration to measure the output value of the balance under single component load loading, establish the relationship between the load and the balance output, and obtain the balance calibration formula. During this process, the axial force and normal force directions are gradually loaded from 0 to 1200 N, and the maximum load in the pitch torque direction is set to 450 Nm, and 16 consecutive loading and unloading operations are carried out. At the same time, in order to verify the feasibility of the F–P sensor designed in this paper in the deformation measurement of sensitive elements and to evaluate the static performance of the optical fiber balance, before the calibration of the optical fiber balance, a resistance strain gauge was installed in the optical fiber balance at the same time to compare the calibration data of the two methods, as shown in Figure 8b.

**Figure 9:**
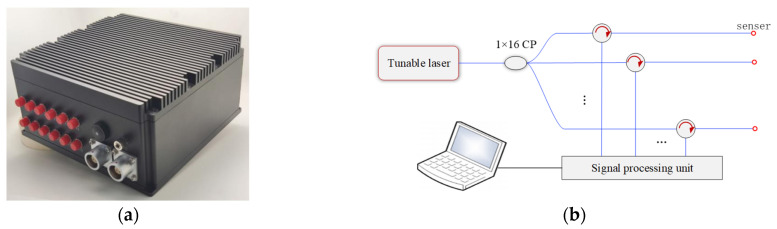
Wavelength demodulator: (**a**) demodulator physical image and (**b**) schematic of the demodulated system.

The wavelength signal is collected using a wavelength demodulator, as shown in Figure 9a, and the system schematic is shown in Figure 9b. The optical fiber spectrum demodulator mainly consists of an optical signal processing board, a tunable laser, a splitter, and a circulator. The laser emits a high-power stable scanning laser, which is divided into 12 optical paths through a 1 × 12 splitter. Each optical path channel is transmitted to the fiber optic spectral sensor through the annular optical path, and the light reflected by the fiber optic spectral sensor enters the annular optical path again for collection by the photodetector. Finally, it is transmitted to the computer through Ethernet. This demodulator can achieve high-speed demodulation at 2 kHz with a wavelength demodulation repeatability accuracy of ±2 pm.

### 3.3. Results and Discussion

**Figure 10:**
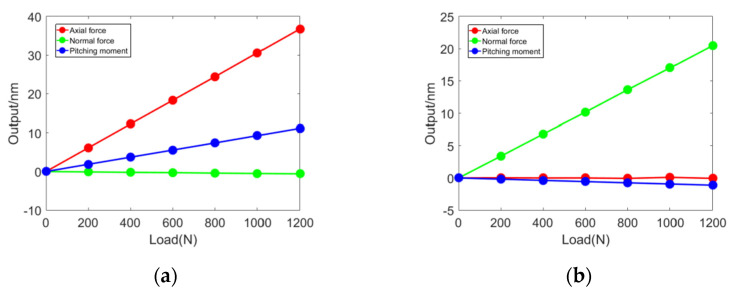

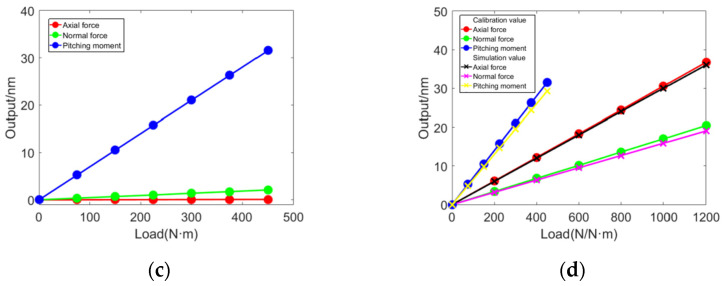
Responses of all aerodynamic components in different load directions: (**a**) *F_A_*, (**b**) *F_N_*, (**c**) *M_Z_*, and (**d**) comparison of experiments and simulations.

The calibration results of the optic fiber balance are shown in Figure 10. Under single-component loading, the linearity between the output of the balance and the load is good, both higher than 0.9993. The comparison between the calibration data of the optic fiber balance and the output values of the simulated balance is shown in Table 5. The error between the output sensitivity of the three components of the optic fiber balance and the simulation value is less than 6%, which is basically consistent with the simulation analysis results. This verifies the feasibility of the design method of the force balance based on F–P displacement measurement.

Secondly, the coupling between different components is also an aspect of the balance that needs to be evaluated. The coupling results between different components of the optic fiber balance are shown in Table 6. Except for the interference of axial force on pitching moment, the interference coefficients between other components are all less than 7.7%, and the interference between some components is less than 1%, which can be basically ignored, indicating that the optic fiber balance has an ideal decomposition of load. In response to the problem of significant interference of axial force on the output of pitching moment, traditional strain balances also have the same problem. This is mainly due to the existence of the loading structure, which causes the loading center to be located 5.74 cm above the balance. Axial force loading will introduce additional pitching moments. From this, it can be seen that when conducting wind tunnel tests, the correlation coefficients in the calibration formula need to be corrected based on the actual position of the model center of gravity.

The static performance indicators of the optic fiber balance are shown in Table 7. Calibration accuracy is the proportion of the standard deviation of the measured output to the full scale, which is the main indicator for evaluating the accuracy of the balance’s force measurement. The calibration accuracy of the three components of the optic fiber balance is 0.1%, 0.13%, and 0.11%, respectively, which meets the requirements of wind tunnel testing (0.5%) and is superior to the calibration accuracy of a traditional strain balance (0.37%).

## 4. Conclusions

This article designs and manufactures a three-component optic fiber balance based on Fabry–Perot displacement measurement for pulse wind tunnel force measurement systems. This F–P displacement sensor is composed of an optical fiber end face and a reflective surface installed on the balance structure, which has the advantages of a simple structure, high sensitivity, and no need for prestressing. Without changing the balance structure or other parameters of the sensor, the sensor can improve the balance sensitivity by changing the probe height. When the probe height is 1 mm or 2 mm, the displacement sensor output is 1.7 times and 2.29 times the actual displacement value of the beam structure, respectively. Based on the simulation analysis results, the sensor design parameters were determined, and a three-component optic fiber balance design method based on eight F–P displacement sensors was proposed, which can achieve temperature self-compensation. Through experimental verification, the temperature self-compensation effect of the optic fiber balance is relatively ideal, and the sensor temperature sensitivity has decreased by 97.4% compared to before compensation. The output sensitivities of each component of the designed three-component optic fiber balance are 0.03 nm/N, 0.017 nm/N, and 0.08 nm/N, respectively. It is basically consistent with the simulation output value, with a linearity higher than 0.9993, which verifies the feasibility of the balance design method based on Fabry–Perot displacement measurement. In terms of performance, the three-component calibration accuracy of the optic fiber balance is better than 0.13%, which meets the requirements of wind tunnel testing and is superior to the traditional strain balance (0.37%). At the same time, the F–P sensor wavelength demodulator used in this article has a sampling rate of 2 kHz. Based on engineering experience, the pulse wind tunnel short-term force measurement test requires measuring system vibration signals of more than five cycles in effective time to compensate for inertial interference. For pulse wind tunnel equipment of the order of 100 milliseconds, the main focus is on interference signal frequencies, usually below 50 Hz. The demodulator’s sampling rate of 2 kHz can effectively capture it.

The high sensitivity and calibration accuracy of optic fiber balances not only improve the data quality of pulse wind tunnel force testing but also verify the ability of optic fiber balances to meet the design requirements of higher stiffness balances. Therefore, from the perspective of larger-scale model testing requirements, an optic fiber balance can improve the stiffness characteristics while ensuring the load resolution ability of the balance in order to solve the problem of reduced natural frequency and dynamic performance of the system caused by increased model mass, which cannot be achieved by traditional strain balance technology. Therefore, developing optic fiber balance will be an important and feasible technical route to expand the capabilities of pulse wind tunnel testing.

## Figures and Tables

**Table 1:** Balance material parameter.

Name	Young’s Modulus (Gpa)	Poisson’s Ratio	Density (kg/m^3^)
F141	187.25	0.27	8000

**Table 2:** Sensor design parameters.

Design Parameter	*d*	*h*	*L*_1_	*L*_2_	*L*_3_	*r*_1_	*r*_2_
Size (mm)	0.073	1	3	5	2	0.5	0.55

**Table 3:** The decoupling formula for its three-component load.

Component	Sensor Combination
*F_A_*	(*FP*1 − *FP*2) + (*FP*3 − *FP*4)
*F_N_*	(*FP*5 − *FP*6) + (*FP*7 − *FP*8)
*M_Z_*	(*FP*5 − *FP*6) − (*FP*7 − *FP*8)

**Table 4:** Temperature compensation test results.

Component	*F_A_*	*F_N_*	*M_Z_*
Sensor without Compensation	~0.46 nm/°C		
Self-Compensation	0.022 nm/°C	0.012 nm/°C	0.031 nm/°C

**Table 5:** Calibration result.

	Output	Simulation Sensitivity	Experimental Sensitivity	Linearity	Residual
Load					
*F_A_*		0.0301 nm/N	0.0306 nm/N	0.9993	0.075782
*F_N_*		0.0161 nm/N	0.0171 nm/N	0.9995	0.063471
*M_Z_*		0.0665 nm/N·m	0.0701 nm/N·m	0.9999	0.006546

**Table 6:** Coupling between different components (“-” means the coupling is less than 1%).

	Output	*F_A_*	*F_N_*	*M_Z_*
Load				
*F_A_*		100%	1.64%	30.3%
*F_N_*		-	100%	5.48%
*M_Z_*		-	6.63%	100%

**Table 7:** Static calibration results of the optic fiber balance.

Component	*F_A_*	*F_N_*	*M_Z_*
Maximum load (Kg)	120	120	45
Maximum output (nm)	36.761	20.468	31.530
Standard deviation	0.035	0.026	0.034
Accuracy	0.1%	0.13%	0.11%

## Data Availability

The data that support the findings of this study are available from the corresponding author upon reasonable request.
